# Comments on *‘FNIP1 regulates adipocyte browning and systemic glucose homeostasis in mice by shaping intracellular calcium dynamics’*

**DOI:** 10.1093/jmcb/mjac033

**Published:** 2022-05-18

**Authors:** Yujing Yin, Dengqiu Xu, Yan Mao, Zhenji Gan

**Affiliations:** State Key Laboratory of Pharmaceutical Biotechnology and MOE Key Laboratory of Model Animal for Disease Study, Division of Spine Surgery, Department of Orthopedic Surgery, Nanjing Drum Tower Hospital, The Affiliated Hospital of Nanjing University Medical School, Jiangsu Key Laboratory of Molecular Medicine, Chemistry and Biomedicine Innovation Center (ChemBIC), Model Animal Research Center, Nanjing University Medical School, Nanjing University, Nanjing 210061, China; State Key Laboratory of Pharmaceutical Biotechnology and MOE Key Laboratory of Model Animal for Disease Study, Division of Spine Surgery, Department of Orthopedic Surgery, Nanjing Drum Tower Hospital, The Affiliated Hospital of Nanjing University Medical School, Jiangsu Key Laboratory of Molecular Medicine, Chemistry and Biomedicine Innovation Center (ChemBIC), Model Animal Research Center, Nanjing University Medical School, Nanjing University, Nanjing 210061, China; State Key Laboratory of Pharmaceutical Biotechnology and MOE Key Laboratory of Model Animal for Disease Study, Division of Spine Surgery, Department of Orthopedic Surgery, Nanjing Drum Tower Hospital, The Affiliated Hospital of Nanjing University Medical School, Jiangsu Key Laboratory of Molecular Medicine, Chemistry and Biomedicine Innovation Center (ChemBIC), Model Animal Research Center, Nanjing University Medical School, Nanjing University, Nanjing 210061, China; State Key Laboratory of Pharmaceutical Biotechnology and MOE Key Laboratory of Model Animal for Disease Study, Division of Spine Surgery, Department of Orthopedic Surgery, Nanjing Drum Tower Hospital, The Affiliated Hospital of Nanjing University Medical School, Jiangsu Key Laboratory of Molecular Medicine, Chemistry and Biomedicine Innovation Center (ChemBIC), Model Animal Research Center, Nanjing University Medical School, Nanjing University, Nanjing 210061, China

Owing to its remarkable benefits on metabolic health and its demonstrated presence in adult humans, beige or ‘brite’ adipocytes hold great promise to combat obesity and metabolic diseases. Delineation of the mechanisms involved in adipocyte ‘beiging’ or ‘browning’ is thus of particular interest. Previous studies of the molecular regulatory pathways that drive the beiging of white adipose tissue (WAT) have centered on the cyclic adenosine monophosphate (cAMP)-related signaling (Wang and Seale, 2016; Cohen and Kajimura, 2021). Acting via the cAMP–protein kinase A pathway, norepinephrine dually stimulates adipocyte lipolysis and the expression of thermogenic genes, such as uncoupling protein 1 (*Ucp1*) and peroxisome proliferator-activated receptor-gamma coactivator-1alpha (*Ppargc1a*) (Inagaki et al., 2016). While the second messenger calcium (Ca^2+^) is fundamental to a wide variety of cellular processes including, but not limited to, muscle contraction, gene transcription, exocytosis, and cellular metabolism in virtually all eukaryotic cells (Arruda and Hotamisligil, 2015), whether and how Ca^2+^ signals orchestrate WAT beiging remains largely unclear, in part because of a limited understanding of the regulatory mechanism that controls intracellular Ca^2+^ dynamics in adipocytes.

In our recent work (Yin et al., 2022), we discovered a previously unrecognized WAT beiging mechanism that targets the folliculin (FLCN) interacting protein 1 (FNIP1)–sarco/endoplasmic reticulum Ca^2+^-ATPase (SERCA)–intracellular Ca^2+^ axis in adipocytes (Figure 1; commented in Bunk and Kazak, 2022). Using multiple Ca^2+^ imaging approaches, together with gain- and loss-of-function strategies, we provided clear data demonstrating that FNIP1 is a crucial regulator of intracellular Ca^2+^ dynamics and negatively regulates WAT beiging. We also uncovered the working mechanisms that FNIP1 binds to and promotes the activity of SERCA, a main Ca^2+^ pump responsible for cytosolic Ca^2+^ removal, thereby dampening Ca^2+^-dependent thermogenic programs. Furthermore, we showed that adipocyte-specific ablation of FNIP1 leads to a broad thermogenic remodeling of WAT with strong effects on whole-body glucose homeostasis and hepatic steatosis.

**Figure 1:**
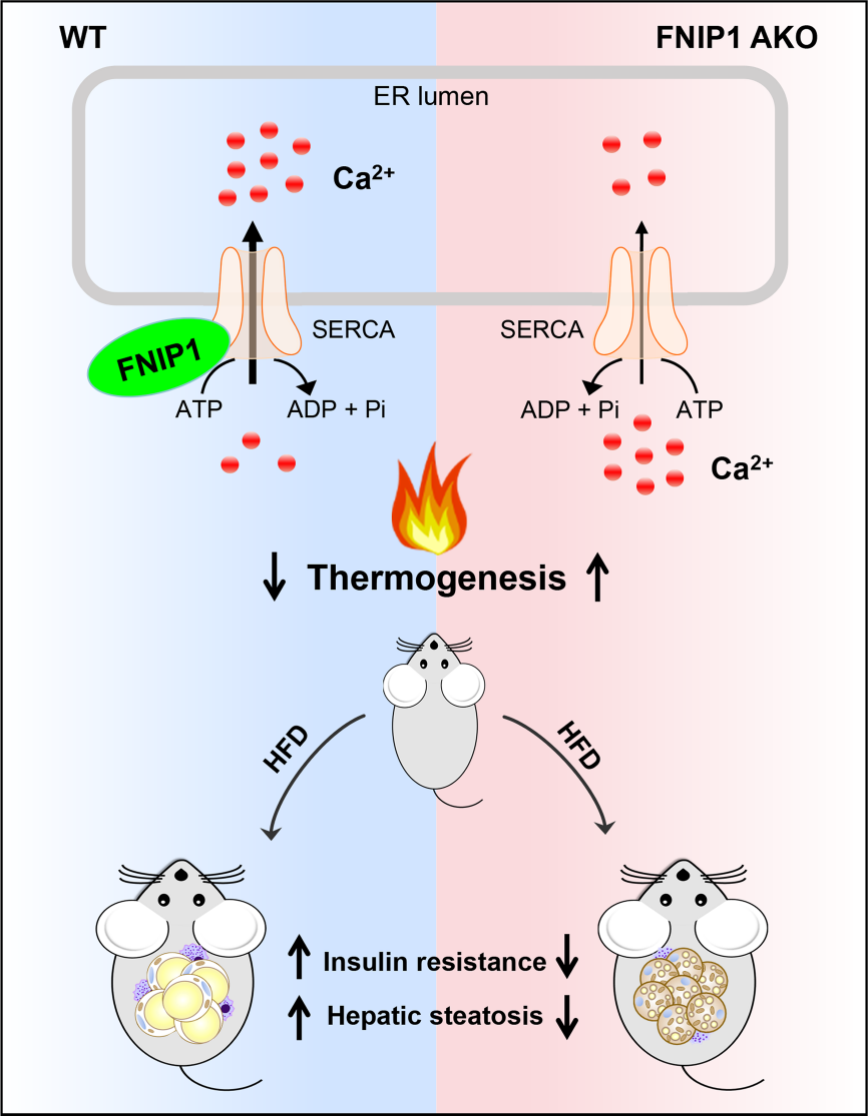
The model of FNIP1 in control of beige adipocyte thermogenesis and metabolic health through modulating SERCA. FNIP1 binds to SERCA to enhance its Ca^2+^ pump activity. This limits intracellular Ca^2+^ dynamics and dampens Ca^2+^-dependent thermogenic programs. Mice with adipocyte-specific ablation of FNIP1 showed enhanced thermogenic remodeling of WAT and were protected against HFD-induced insulin resistance and hepatic steatosis.

In this study, we set to investigate the intracellular Ca^2+^ dynamics in association with WAT beiging by the employment of GCaMP6f reporter mice, a mouse model that allowed us to monitor Ca^2+^ activity in adipose tissue *in vivo*. We found that dynamic changes of intracellular Ca^2+^ are required for the thermogenic gene program in white adipocytes upon thermogenic stimuli. The endoplasmic reticulum (ER) serves as the main Ca^2+^ store that determines intracellular Ca^2+^ homeostasis. Among the ER membrane-localized Ca^2+^ channels, SERCA is the solely Ca^2+^ influx pump that has been shown to fine-tune intercellular Ca^2+^ dynamics. By performing immunoprecipitation and mass spectrometry analysis, we identified that SERCA2 is able to interact and precipitate with FNIP1, an adaptor protein originally identified through its interaction with FLCN and AMP-activated protein kinase (AMPK) (Baba et al., 2006). We then performed a series of cellular assays to demonstrate that FNIP1 acts as a positive regulator of SERCA in adipocytes, and binding of FNIP1 promotes both ATP hydrolysis and Ca^2+^ transport of the SERCA pump, leading to a faster removal of cytosol Ca^2+^. As such, in FNIP1 knockout (KO) adipocytes, the reuptake of cytosolic Ca^2+^ into the ER lumen mediated by SERCA is reduced, resulting in elevated Ca^2+^ levels and an increased duration of Ca^2+^ transients in the cytosol upon external stimuli. More importantly, we showed that, functionally, loss of adipocyte FNIP1 in mice results in enhanced thermogenic remodeling in WAT. This improves systemic glucose homeostasis and prevents hepatic steatosis when mice are fed a high-fat diet (HFD).

We observed that FNIP1 deficiency in adipocytes triggers a robust UCP1^+^ beige adipocyte formation and mitochondrial biogenesis in the absence of cold exposure or β-adrenergic stimulation. Therefore, FNIP1 acts as an endogenous ‘brake’ to inhibit adipocyte beiging. Intriguingly, our results also indicate that FNIP1 suppression of thermogenic programs occurs in a depot-selective manner, the reason for which is not clear. This could reflect the intrinsic differences between beige adipocytes and brown adipocytes that affect the adaptive range of thermogenic remodeling.

While we have demonstrated previously that FNIP1 acts through AMPK but not mammalian target of rapamycin (mTOR) complex 1 signaling to regulate mitochondrial function in the skeletal muscle (Xiao et al., 2021), interestingly, we did not observe a change in either AMPK or mTOR signaling in both FNIP1 KO primary adipocytes and FNIP1 adipocyte-specific KO (AKO) adipose tissues. These results suggest that the regulations of metabolic regulators by FNIP1 are context-specific or cell type-specific. We clearly showed that FNIP1 binds to and promotes the activity of SERCA. We also examined the endogenous FNIP1 and SERCA interaction in adipose tissue after adrenergic stimulation. The results were interesting and consistent with the proposed mechanistic model. We found that CL316,243 stimulation significantly reduced the interaction between endogenous FNIP1 and SERCA in WAT.

Previously published studies have revealed that FNIP1 could actually act as a co-chaperone that regulates the chaperone function of Hsp90 (Woodford et al., 2016). We found that the N-terminal region of FNIP1 binds to the ATP hydrolysis site of the SERCA pump. Moreover, we observed that SERCA protein was stabilized in cells expressing FNIP1. Based on our data and the published literatures, it is possible that FNIP1 also functions as a co-chaperone of Hsp90 that regulates SERCA protein stability in adipocytes.

Notably, a recent study has shown that enhanced Ca^2+^ cycling in beige fat via the SERCA2–RyR pathway can promote a UCP1-independent thermogenesis (Ikeda et al., 2017). Whereas UCP1 and mitochondria were strongly induced by FNIP1 deletion, thus suggesting a UCP1-dependent thermogenesis in FNIP1 AKO inguinal WAT, our results did not determine whether this FNIP1 regulatory pathway is involved in UCP1-dependent or UCP1-independent beige adipocyte thermogenesis. Obviously, these are not mutually exclusive roles.

In summary, our recent work suggests that FNIP1 acts as a negative regulator of beige adipocyte thermogenesis to control systemic glucose homeostasis through modulating SERCA (Yin et al., 2022). Linking intracellular Ca^2+^ dynamics to adipocyte beiging is of interest, given that it could be an appealing strategy for the treatment of metabolic disorders. This raises several interesting questions. (i) What are the mechanisms by which the intracellular Ca^2+^ signals activate the thermogenic program? (ii) How is the interaction between FNIP1 and SERCA regulated under the physiological setting? (iii) Does this FNIP1–SERCA–intracellular Ca^2+^ pathway represent a broader metabolic regulatory network in other organs? (iv) Will it be possible to exploit such FNIP1–intracellular Ca^2+^ signals as treatment strategies?

*[This work was supported by grants from the National Natural Science Foundation of China (31922033, 91857105, 32071136, 32100922, and 32100942), the Natural Science Foundation of Jiangsu Province (BK20170014 and SWYY-002), the China Postdoctoral Science Foundation (2021M691524), and the Fundamental Research Funds for the Central Universities (021414380511).]*

## References

[bib1] Arruda A.P. , Hotamisligil G.S. (2015). Calcium homeostasis and organelle function in the pathogenesis of obesity and diabetes. Cell Metab. 22, 381–397. 26190652 10.1016/j.cmet.2015.06.010 PMC4558313

[bib2] Baba M. , Hong S.-B., Sharma N. et al. (2006). Folliculin encoded by the BHD gene interacts with a binding protein, FNIP1, and AMPK, and is involved in AMPK and mTOR signaling. Proc. Natl Acad. Sci. USA 103, 15552–15557. 17028174 10.1073/pnas.0603781103 PMC1592464

[bib3] Bunk J. , Kazak L. (2022). Calcium burns beige. J. Exp. Med. 219, e20220382. 35412554 10.1084/jem.20220382 PMC9123248

[bib4] Cohen P. , Kajimura S. (2021). The cellular and functional complexity of thermogenic fat. Nat. Rev. Mol. Cell Biol. 22, 393–409. 33758402 10.1038/s41580-021-00350-0 PMC8159882

[bib5] Ikeda K. , Kang Q., Yoneshiro T. et al. (2017). UCP1-independent signaling involving SERCA2b-mediated calcium cycling regulates beige fat thermogenesis and systemic glucose homeostasis. Nat. Med. 23, 1454–1465. 29131158 10.1038/nm.4429 PMC5727902

[bib6] Inagaki T. , Sakai J., Kajimura S. (2016). Transcriptional and epigenetic control of brown and beige adipose cell fate and function. Nat. Rev. Mol. Cell Biol. 17, 480–495. 27251423 10.1038/nrm.2016.62 PMC4956538

[bib7] Wang W.S. , Seale P. (2016). Control of brown and beige fat development. Nat. Rev. Mol. Cell Biol. 17, 691–702. 27552974 10.1038/nrm.2016.96 PMC5627770

[bib8] Woodford M.R. , Dunn D.M., Blanden A.R. et al. (2016). The FNIP co-chaperones decelerate the Hsp90 chaperone cycle and enhance drug binding. Nat. Commun. 7, 12037. 27353360 10.1038/ncomms12037 PMC4931344

[bib9] Xiao L. , Liu J., Sun Z. et al. (2021). AMPK-dependent and -independent coordination of mitochondrial function and muscle fiber type by FNIP1. PLoS Genet. 17, e1009488. 33780446 10.1371/journal.pgen.1009488 PMC8031738

[bib10] Yin Y. , Xu D., Mao Y. et al. (2022). FNIP1 regulates adipocyte browning and systemic glucose homeostasis in mice by shaping intracellular calcium dynamics. J. Exp. Med. 219, e20212491. 35412553 10.1084/jem.20212491 PMC9008465

